# Rapid evolution of mammalian X-linked testis microRNAs

**DOI:** 10.1186/1471-2164-10-97

**Published:** 2009-03-04

**Authors:** Xuejiang Guo, Bing Su, Zuomin Zhou, Jiahao Sha

**Affiliations:** 1 Laboratory of Reproductive Medicine, Department of Histology and Embryology, Nanjing Medical University, Nanjing 210029, PR China; 2 State Key Laboratory of Genetic Resources and Evolution, Kunming Institute of Zoology and Kunming Primate Research Center, Chinese Academy of Sciences, Kunming 650223, Yunnan, PR China

## Abstract

**Background:**

MicroRNAs (miRNAs), which are small, non-coding RNAs approximately 21-nucleotides in length, have become a major focus of research in molecular biology. Mammalian miRNAs are proposed to regulate approximately 30% of all protein-coding genes. Previous studies have focused on highly conserved miRNAs, but nonconserved miRNAs represent a potentially important source of novel functionalities during evolution.

**Results:**

An analysis of the chromosome distribution of miRNAs showed higher densities of miRNAs on the X chromosome compared to the average densities on autosomes in all eight mammalian species analyzed. The distribution pattern did not, however, apply well to species beyond mammals. In addition, by comparing orthologous human and mouse miRNAs, we found that X-linked miRNAs had higher substitution rates than autosomal miRNAs. Since the highest proportion of X-linked miRNAs were found in mouse testis, we tested the hypothesis that testis miRNAs are evolving faster on the X chromosome than on autosomes. Mature X-linked testis miRNAs had an average substitution rate between mouse and human that was almost 25-fold higher than mature testis miRNAs on autosomes. In contrast, for mature miRNAs with precursors not expressed in testis, no significant difference in the substitution rate between the X chromosome and autosomes was found. Among mammals, the rapid evolution of X-linked testis miRNAs was also observed in rodents and primates.

**Conclusion:**

The rapid evolution of X-linked testis miRNAs implies possible important male reproductive functions and may contribute to speciation in mammals.

## Background

MicroRNAs (miRNAs) are families of small, non-coding RNAs that are approximately 21-nucleotide in length. Non-coding RNAs have emerged as key post-transcriptional regulators of gene expression in metazoans and plants, and thus have become a major focus of research. By base pairing to mRNAs, miRNAs mediate translational repression or mRNA degradation [1]. Bioinformatics prediction indicates that mammalian miRNAs may regulate ~30% of all protein-coding genes [2]. Functional studies indicate that miRNAs participate in the regulation of almost every cellular process investigated to date [3-7].

Until now, most research has focused on highly conserved miRNAs. Most current computational methods for the prediction of miRNA genes rely heavily on phylogenetic conservation of sequences, but nonconserved miRNAs represent a potentially important source of novel functions during evolution. In this study, we analyzed the genomic distribution of miRNAs and found higher densities of miRNAs on the mammalian X chromosome. Evolutionary analysis of miRNAs suggests rapid evolution of X-linked miRNAs, which may be related to their function in testis.

## Results

### Distribution of miRNAs on chromosomes in mammalian and other species

**Figure 1:**
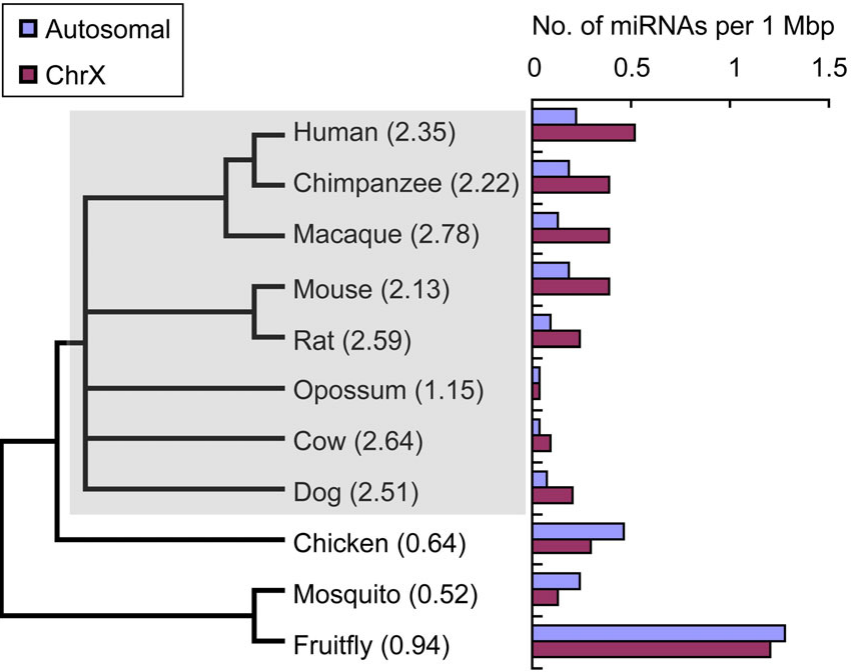
**Density distribution of miRNAs across chromosomes among species**. Densities of miRNAs on the X chromosome and autosomes are shown for different species, except for chicken, where the Z chromosome is used instead of X. Densities are shown as number of miRNAs per megabase of DNA. The ratios of densities between the X chromosome and autosomes are shown in brackets. Mammals in the species tree on the left are shaded in grey.

Using miRNA data from the miRBase database we calculated the densities of miRNAs on chromosomes and found no miRNA on the Y chromosome in any species. Therefore, for subsequent distribution analysis of miRNAs on sex chromosomes, only the X chromosome was considered. By comparing the densities of miRNAs on the X chromosome and the average densities on autosomal chromosomes, we found a higher density of miRNAs on X chromosomes in mammalian species. In seven of eight mammalian species, the densities were greater than two-fold those on autosomes (Fig. 1). A paired Student's t-test confirmed significantly higher densities of miRNAs on X chromosome than those on autosomal chromosomes, across mammalian species (p < 0.01).

The XX/XY system is one of the most common sex-determination systems and is found in the vast majority of mammals. To better examine the differences of miRNA densities between X chromosomes and autosomes, we calculated the density distributions in species beyond mammals, and found a ratio of 0.52 in mosquito and 0.94 in fruitfly. In addition, we calculated the distribution of miRNAs in chicken, which has the ZW sex-determination system, in which females have two different kinds of chromosomes (ZW), and males have two of the same kind (ZZ). We found no miRNA on the W chromosome, and a ratio of densities between the Z chromosome and autosomes of 0.64, a value less then 1, meaning that chicken has lower miRNA densities on the sex chromosome. Thus, it seems that mammals have higher densities of miRNAs on the X chromosome, but this phenomenon does not extend to all species. In fact, species other than mammals may even have reversed density distributions.

### Rapid evolution of X-linked miRNAs in mammals

**Figure 2:**
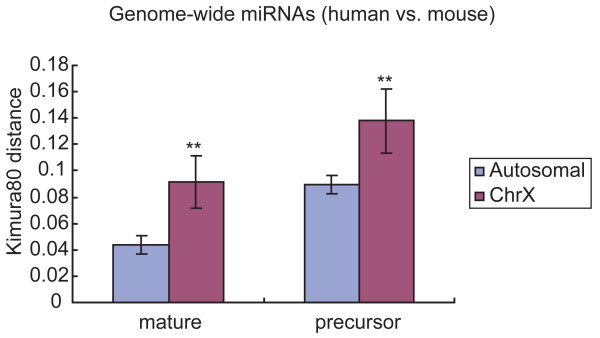
**Comparison of substitution rates for genome-wide miRNAs between human and mouse by chromosomes**. Substitution rate differences of genome-wide mature miRNAs and precursor miRNAs between the X chromosome and autosomes are shown. Substitution rates were calculated from orthologous sequences of human and mouse using the Kimura 2-parameter model. All comparisons showed significant differences (**, p < 0.01).

The precursor miRNA substitution rates between human and mouse were calculated using the Kimura 2-parameter model. Comparison of substitution rates showed significantly higher rates for X-linked than for autosomal precursor miRNAs (p = 0.0054) (Fig. 2). The average substitution rates were 0.138 and 0.089, respectively. In addition, we calculated the substitution rates for mature miRNAs, which are the functional part of precursor miRNAs. The substitution rates for X chromosome and autosomes were significantly different (p = 6.9E-5), at 0.091 and 0.043, respectively. The ratio of mature miRNAs between the X chromosome and autosomes (2.1 fold) was greater than that of precursors (1.5 fold).

### Tissue distribution of X-linked miRNAs in mouse

**Figure 3:**
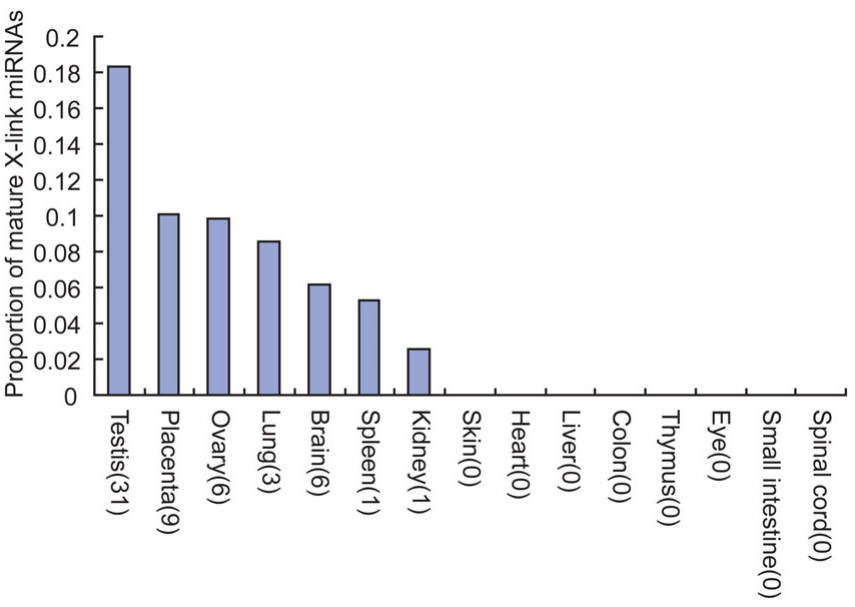
**Proportion of mature X-linked mouse miRNAs by tissues**. miRNA data are from the mammalian microRNA expression atlas based on the small RNA library sequencing by Landgraf et al. [8]. The numbers of X-linked miRNAs cloned in corresponding tissues are shown in brackets.

Landgraf et al. [8] constructed a mammalian microRNA expression atlas based on a multi-laboratory effort to sequence small RNA libraries. We used the data to analyze the expression of X-linked mature miRNAs across different tissues in mouse. For 15 different mouse tissues, we found the highest number of X-linked mature miRNAs in testis. The proportion of X-linked mature miRNAs was also highest in testis. For the 169 mature miRNAs identified from mouse testis whose genomic loci were mapped, 31 (18.3%) of them were from the X chromosome. (Fig. 3)

### Rapid evolution of mammalian X-linked testis-expressedmiRNAs

**Figure 4:**
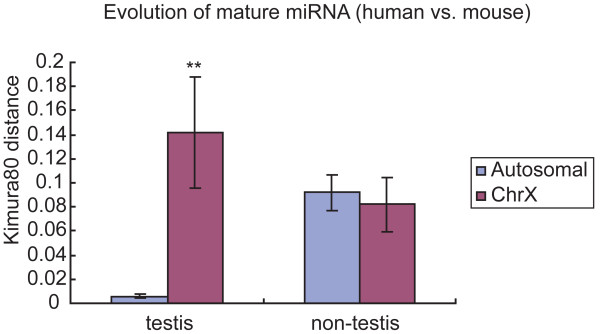
**Comparison of substitution rates in testis and non-testis mature miRNAs across chromosomes**. Differences in substitution rates of mature miRNAs on the X chromosome and autosomes are shown. Substitution rates were calculated from orthologous sequences of human and mouse using the Kimura 2-parameter model. For non-testis mature miRNAs, miRNAs with precursors not expressed in mouse testis were used. Comparisons with significant differences are shown with ** (p < 0.01).

Because the testis contains the highest proportion of X-linked miRNAs in mouse, we separated miRNAs into two genome-wide classes: those with evidence of expression in mouse testis, and those without. Presently, mouse is the species with most comprehensive data of miRNAs in testis. In addition to the Landgraf et al.'s miRNA atlas[8], Ro et al. [9] also cloned mouse testis miRNAs. To achieve a higher coverage of testis-expressed miRNAs, both two datasets were used. For a total of 609 mature miRNAs, 548 were unique to a precursor and used for analysis, and of these, 181 were annotated as expressed in testis. Comparison of 145 testis-expressed mature miRNAs with their human orthologs showed a significantly higher substitution rate for X-linked mature miRNAs than for autosomal mature miRNAs (Kimura80 distance: 0.1417 and 0.0056, respectively; ratio: 25.2, p-value < 0.01) (Fig. 4). The difference for testis-expressed mature miRNAs was almost 25-fold, so non-testis mature miRNAs were analyzed for similar differences. For 157 mature miRNAs with precursors not expressed in testis and with orthologs in human, the substitution rate difference between the X chromosome and autosomes was not significantly different (Kimura80 distance: 0.082 and 0.091, respectively; ratio: 0.89, p-value > 0.05). Thus, the higher substitution rates seen in testis-expressed mature miRNAs correlated with their testis expression (Fig. 4).

**Figure 5:**
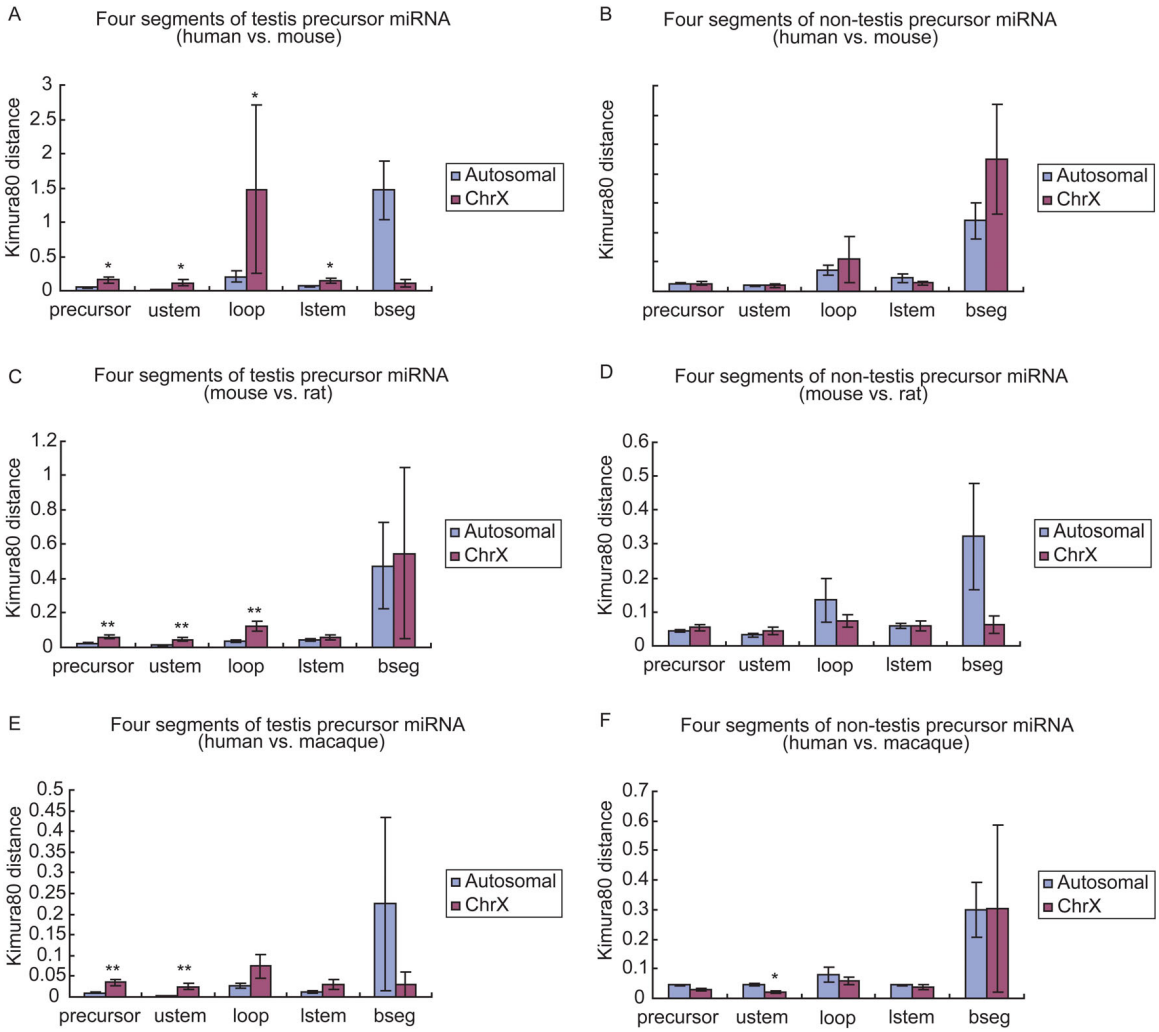
**Comparison of substitution rates for testis and non-testis precusor miRNAs across chromosomes**. Differences in substitution rates for precursor miRNAs on the X chromosome and autosomes, as well as their four parts, were compared. For testis precursor miRNAs, substitution rate comparisons were performed for human and mouse orthologs (A), for mouse and rat (C), and for human and macaque (E). The same analysis was performed for precursor miRNAs not expressed in testis (B, D and F, respectively). The names used are: precursor, precursor miRNA; ustem, upper stem region; loop, terminal loop; bseg, basal segments. Significant differences are indicated with * (p < 0.05) or ** (p < 0.01).

To better characterize the evolution of testis-expressed miRNAs, we compared the testis-expressed precursor miRNAs and found their substitution rates were also higher on the X chromosome than on autosomes (p-value < 0.05). After dividing the precursor into four parts: terminal loop, upper stem region, lower stem region and basal segments, we found significant differences (p < 0.05) for the terminal loop, upper stem region and lower stem region. For miRNAs not expressed in testis, neither the entire precursor nor any of the four parts showed a significant difference between the X chromosome and autosomes (Fig. 5A–B).

We did additional analyses to see if the phenomenon of rapid evolution of X-linked testis miRNAs existed in the primate and rodent branches of mammals. For orthologous miRNA sequences between mouse and rat, X-linked testis precursor miRNAs evolved faster than those on autosomes, with a significant difference (p < 0.01). The upper stem regions and loops of testis precursor miRNAs also showed higher substitution rates on X chromosome than on autosomes (p < 0.01). In primates, the Kimura 2-parameter substitution rates between human and macaque were higher for X-linked testis precursor miRNAs than for autosomal testis ones (p < 0.01), and the substitution rates were also higher in the upper stem regions for X-linked testis miRNAs (p < 0.01). For miRNAs not expressed in testis, the Kimura 2-parameter distances of precursor miRNAs in both rodents and primates showed no difference between the X chromosome and autosomes (Fig. 5C–F). The prediction of orthologous sequences used is given in Methods [see Additional file 1]. Testis expression data for human and macaque were from the human miRNA tissue distribution data in the miRNA atlas by Landgraf et al. [8].

### Target gene analysis of X-linked miRNAs

Target genes of X-linked miRNAs, in both testis and non-testis, were predicted by TargetScan, which uses sequence conservation information across mammals. Although some X-linked miRNAs have non-conserved seed sequences, for many miRNAs, the prediction of non-conserved targets will yield substantial false positives that could hinder meaningful functional comparison between target gene sets. Thus, only X-linked miRNAs with conserved sequences at nucleotides 2–8 were used for target gene analysis. Using this criterion, six X-linked testis miRNAs and 16 X-linked non-testis miRNAs were analyzed. Comparison of enriched biological process terms between target gene sets of X-linked testis miRNAs and X-linked non-testis miRNAs showed that the most enriched four terms exclusively in target gene set of X-linked testis miRNAs were cell cycle, cell cycle process, regulation of progression through cell cycle and regulation of cell cycle [see Additional file 2], which indicated the important roles of X-linked testis miRNAs in the cell cycle.

## Discussion

The mammalian sex chromosomes arose from autosomal progenitors approximately 300 million years ago. Before then, sex determination probably relied on environmental cues such as egg incubation temperature, as is the case in many extant reptiles [10]. Ross et al. [11] confirmed that much of the long arm of the human X is homologous to the short arm of chicken chromosome 4, whereas most of the short arm of human X matches a stretch of chicken chromosome 1. The bird sex chromosomes Z and W, on the other hand, show homology to human chromosome 9. These observations demonstrate the independent origins of genetic sex determination in mammals and birds. Furthermore, comparison of the human and dog X chromosomes indicates that the gene order largely represents that of the ancestral X. There is also remarkable synteny of the X chromosome between human, mouse and rat [11]. Distribution analysis of miRNAs across chromosomes showed that densities of miRNAs on X were higher than the average densities on autosomes for eight mammals analyzed, including human, mouse, rat and dog. However, this difference between chromosomes does not necessarily exist in species outside mammals. The density of miRNAs on the mosquito X chromosome was about half that of autosomes. For chicken, whose sex chromosome has origin different from mammalian sex chromosome, the density of miRNAs on the Z chromosome was lower than that on autosomes. It should be noted that miRNAs from some of the analyzed species such as cow, dog and chicken, have not been fully identified, which may cause potential bias in miRNA density analysis. As more comprehensive miRNA databases are available for the species analyzed here, and others, it will be possible to test whether this is a widely existing phenomenon. According to current data, however, the higher density of miRNAs on X implies that the X-linked miRNAs may have certain X-related properties or functions in mammals.

Analysis of substitution rates of genome-wide orthologous precursor and mature miRNAs between human and mouse showed that the rates were higher on the X chromosome. With the available comprehensive testis miRNA data in mouse and the hints that mouse testis contains the largest number and highest proportion of X-linked mature miRNAs, we found the substitution rate difference between X and autosomes was evident only for testis-expressed mature and precursor miRNAs. The difference disappeared for mature and precursor miRNAs not expressed in testis. By dividing precursor miRNAs into four parts, differences were observed for upper stem parts, terminal loops and lower stem parts. In addition, the phenomenon of faster evolution for X-linked testis precursor miRNAs was also seen in rodents and primates by comparing orthologous miRNA sequences between mouse and rat, and between human and macaque. Differences for upper stem parts of precursor miRNA were significant in all comparisons between X-linked testis miRNAs and autosomal testis miRNAs. Upper stems are functional parts of precursor miRNAs. Their rapid evolution in X-linked testis precursor miRNA implies that they may play important roles in testis function. Chromosomal location alone does not explain the differences in substitution rate.

Sex chromosomes and sex-linked genes have been a central focus of research in many areas of evolutionary biology, and there are conflicting evidences as to whether the evolutionary rate of the X chromosome is accelerated or reduced compared with that of autosomes [12]. One theory is that there are greater selective constraints on the X-linked genes than on autosomal genes, as any recessive deleterious mutations will be expressed in males and may be subject to stronger purifying selection [13]. However, there are certain conditions where genes on the X chromosome can evolve faster than those on autosomes. If the majority of new mutations are beneficial and are at least partially recessive, haploid expression in the heterogametic sex will result in higher rates of sequence divergence [14]. Two studies support the second scenario of faster X evolution due to an increased likelihood of positive selection or a selective sweep of beneficial recessive mutations on the X chromosome. First, reduced polymorphism on the X chromosome has been reported in *Drosophila simulans*, suggesting that a form of positive selection may be acting on sex chromosomes [15]. Second, a recent study of X-linked and sex-biased protein-coding gene evolution in Drosophila provided the evidence for increased adaptive evolution of X-linked genes, consistent with a fast-X effect. This study also revealed a strong fast-X effect for male-biased genes and a weak, but significant, fast-X effect for unbiased genes [16]. These results suggest the frequent occurrence of recessive beneficial mutations. Although many similar studies have been done on the evolution of protein-coding genes, little has been reported for non-coding genes, especially the important miRNA genes.

In this study, high substitution rates between orthologous X-linked miRNAs from human and mouse were observed, and a general pattern of rapid evolution was shown for mammalian X-linked testis-expressed miRNA genes. Additionally, Zhang et al. [17] have reported that three human X-linked testis miRNAs, miR-513-1, miR-508 and miR-510, have an excess of substitutions over neutrality and are under positive selection. This phenomenon is consistent with and supported by Rice's hypothesis [18], which states that because males carry a single X chromosome, any recessive allele arising on the X that gives males a reproductive advantage is immediately available for positive selection [19]. Thus, there is a possibility that X-linked testis miRNA may have important reproductive functions in males and their fast evolution may contribute to mammalian speciation.

Spermatogenesis in testis involves massive and continuous cell division, from mitosis of spermatogonia to meiosis of spermatocytes. This leads to approximately 40 million sperm per testis daily in a sexually mature mouse [20]. Gene ontology annotation of the target genes of X-linked miRNAs showed that the most enriched terms exclusively in targets of testis X-linked miRNAs were related to the cell cycle process. The enrichment of these target genes by X-linked testis miRNAs implied that these testis miRNAs might participate in regulation of mitosis of spermatogonia and/or meiosis of spermatocytes and consequently be important for the regulation of spermatogenesis. In a recent research report, male mice lacking *Dicer1*, which is required for miRNA processing, specifically in germ cells were subfertile. During the initial wave of spermatogenesis, testes were approximately 50% smaller than those of wild type littermates, had a decreased number of germ cells and contained many Sertoli-cell only tubules [21]. The loss of X-linked testis miRNAs with predicted functions in cell cycle regulation may contribute to these defects.

## Conclusion

We have shown that testis-expressed miRNAs on the X-chromosome are highly diverged compared with those on autosomes for mouse, rat, human and macaque comparisons. Sexual selection can influence sex gene-pool evolution by broad-sense sexual selection and may not be limited to secondary sexual traits and mating behavior [22]. The hemizygosity of the X chromosome, X-autosomal interaction, the higher proportion of X-linked miRNAs in testis, and stronger antagonistic and/or adaptive sexual selection in the male, can all collectively drive evolution of testis miRNAs that are X-linked, at a faster rate than those that are autosomal. In the future, it would be of great interest to work out the developmental pathways by which these miRNAs function and the biological significance of their rapid pace of evolution.

## Methods

### Comparison of miRNA densities on the X chromosome andautosomes

The genome coordinates of miRNAs on chromosomes were from miRBase Release 12.0 [23]. Densities of chromosomal miRNAs were calculated by dividing the number of miRNAs on the X chromosome or all autosomes by the length of nucleotides on the corresponding chromosomes for all species analyzed except chicken, for which the Z chromosome was used instead of X chromosome. Species whose genomic sequences are not assembled into chromosomes in the public databases were not used. Species used in the analysis were human (*Homo sapiens*, NBCI36), chimpanzee (*Pan troglodytes*, PANTRO2.1), macaque (*Macaque mulatta*, MMUL1.0), mouse (*Mus musculus*, NCBIM37), rat (*Rattus norvegicus*, RGSC 3.4), opossum (*Monodelphis domestica*, MONDOM5.0), cow (*Bos Taurus*, BTAU4.0), dog (*Canis familiaris*, CanFam 2.0), chicken (*Gallus gallus*, WASHUC2), mosquito (*Anopheles gambiae*, AgamP3), and fruitfly (*Drosophila melanogaster*, BDGP 5.0).

### Orthologous miRNA prediction

The mouse miRNA data used were from miRBase Release 11.0 [23]. Rat orthologs of mouse miRNAs were predicted using MULTIZ, a sequence alignment program that accurately aligns vertebrate genomes in the ncRNA regions. MULTIZ assumes that all matching segments occur in the same order and orientation in the given sequences by synteny. [24,25]. To further improve the quality of ortholog prediction, we kept only the predicted rat miRNAs that could be mapped to the same miRNA in mouse, that is, the rat and mouse orthologs that reciprocally mapped to each other using MULTIZ. Predicted rat miRNAs were filtered with thresholds of RNA secondary folding energy at -20 kcal/mole (mfold) and 80% sequence length coverage [26,27]. The human orthologs of mouse miRNAs were predicted using the same strategy, as was prediction of macaque orthologs of human miRNAs except that blastz genome sequence alignment [28] was used to map predicted macaque ortholog sequences to human as no pre-computed MULTIZ alignments for macaque was available. The human miRNA data used were from miRBase Release 11.0. The UCSC genome databases [29] were used for sequence analysis, including the Human Mar. 2006 (hg18), the Rhesus Jan. 2006 (rheMac2), the Rat Nov. 2004 (rn4) and the Mouse Jul. 2007 (mm9) assemblies. The pre-computed genome sequence alignments were from the UCSC genome browser [30].

### Calculation of the substitution rates of miRNAs betweenspecies

The orthologous sequences of two species were aligned by ClustalX. The Kimura 2-parameter model was used to calculate substitution rates (Kimura80 distance) of the precursor miRNAs and mature miRNAs [31]. For precursor miRNAs, the secondary structure was predicted by mfold [27]. The pre-miRNA sequences were divided into terminal loop, upper stem, lower stem and basal segments as described before [32]. The substitution rates of each part were also calculated using the Kimura 2-parameter model.

### miRNA expression datasets

The multi-tissue mouse miRNA expression data were from a mammalian miRNA expression altas based on small RNA library sequencing [8]. Additional mouse testis miRNA profiling data [9] were used to improve the comprehensiveness of testis miRNA expression. We determined the expression of a precursor miRNA in a given tissue according to its mature miRNAs. A precursor miRNA was determined to be expressed in testis when expression of a corresponding mature miRNA unique to this precursor was found. If a precursor miRNA had only one mature miRNA expressed in the testis, but it is not unique to this precursor, expression was treated as indeterminant.

### Target prediction and gene ontology analysis

Target gene analysis was performed using Targetscan 4.2 server [2], which uses nucleotides 2–8 of an miRNA as input. It uses TargetScanS to predict biological targets of annotated miRNAs by searching for the presence of 7- and 8-mer sites that match the seed region of each miRNA. A gene with conserved sites in human, mouse, rat and dog was considered a target. Only miRNAs with conserved nucleotides 2–8 in human, mouse, rat and dog was used for analysis; miRNAs without unique nucleotides at 2–8 were excluded because TargetScan cannot differentiate target genes for these miRNAs.

Gene ontology enrichment analysis of target genes was performed using the program: DAVID 2008 [33], a Web-based, client/server application that allows users to access a relational database of functional annotation. A biological process term was considered to be significantly enriched if the p-value was less than 0.05 (Benjamini-Hochberg procedure for multiple correction).

### Statistical Analysis

A Kolmogorov-Smirnov Z test was performed to detect significant differences of the substitution rates (Kimura80 distances) between chromosomes. For comparison of miRNA densities between the X chromosome and autosomes, a paired Student's t-test was used. A p-value less than 0.05 was considered statistically significant. All data are given as mean ± standard error of the mean (SEM).

## Abbreviations

miRNA: microRNA; SEM: standard error of the mean.

## Authors' contributions

XG, BS, ZZ and JS designed the experiments. XG performed the analyses. XG wrote the manuscript, which was read and approved by all authors.

## Supplementary Material

Additional file 1 Genomic coordinates of predicted orthologous sequences of miRNA in different species. Excel spreadsheet presenting homology data on the mouse:human, mouse:rat, and human:macaque putative orthologs together with RNA secondary folding energies. Click here for file

Additional file 2 Gene ontology enrichment exclusively in target genes of X-linked testis miRNAs. Excel spreadsheet presenting gene ontology enrichment exclusively in target genes of X-linked testis miRNAs. Click here for file
